# How enzymes make the right choice during biosynthesis

**DOI:** 10.7554/eLife.111042

**Published:** 2026-04-08

**Authors:** Ana S Ramírez, Camilo Perez

**Affiliations:** 1 Department of Biochemistry and Molecular Biology, University of Georgia Athens United States; 2 Complex Carbohydrate Research Center, University of Georgia Athens United States

**Keywords:** hyaluronan, glycobiology, polysaccharide, extracellular matrix, enzyme mechanism, None

## Abstract

The biosynthesis of an important biopolymer called hyaluronan requires an enzyme that discriminates between two different substrates.

**Related research article** Stephens Z, Karasinska J, Zimmer J. 2026. Insights into substrate binding and utilization by hyaluronan synthase. *eLife* **14**:RP109624. doi: 10.7554/eLife.109624.

From prokaryotes to eukaryotes, cell surfaces are decorated with biopolymers that are essential for a wide range of processes, including cellular defense, environmental adaptation, intercellular communication, and tissue and organ structure (Frantz et al., 2010; Whitfield et al., 2020). These biopolymers are chemically complex, often consisting of thousands of repeating units. For example, hyaluronan – a biopolymer that plays vital structural roles in connective tissue, vascular tissue and cartilage in vertebrates – consists of more than 10,000 repeating units made of glucuronic acid (GlcA) and N-acetylglucosamine (GlcNAc) (Cowman et al., 2015; Litwiniuk et al., 2016).

Hyaluronan biosynthesis is a multistep process that involves alternately binding one of two substrates, UDP-GlcA and UDP-GlcNAc, to the catalytic site of an enzyme called hyaluronan synthase, which is embedded in the plasma membrane of the cell (Figure 1; Górniak et al., 2026; Maloney et al., 2022). This enzyme is also responsible for translocating the hyaluronan polymer through the plasma membrane, which allows it to extend into the extracellular space (DeAngelis and Zimmer, 2023). Hyaluronan synthases can be classified as processive (Class 1) enzymes or as distributive (Class 2) enzymes. Processive hyaluronan synthases can elongate either the non-reducing end of the polymer (Class 1-NR enzymes) or the reducing end (Class 1 R enzymes). On the other hand, distributive enzymes are only able to elongate the non-reducing end (Blackburn et al., 2018).

**Figure 1.**
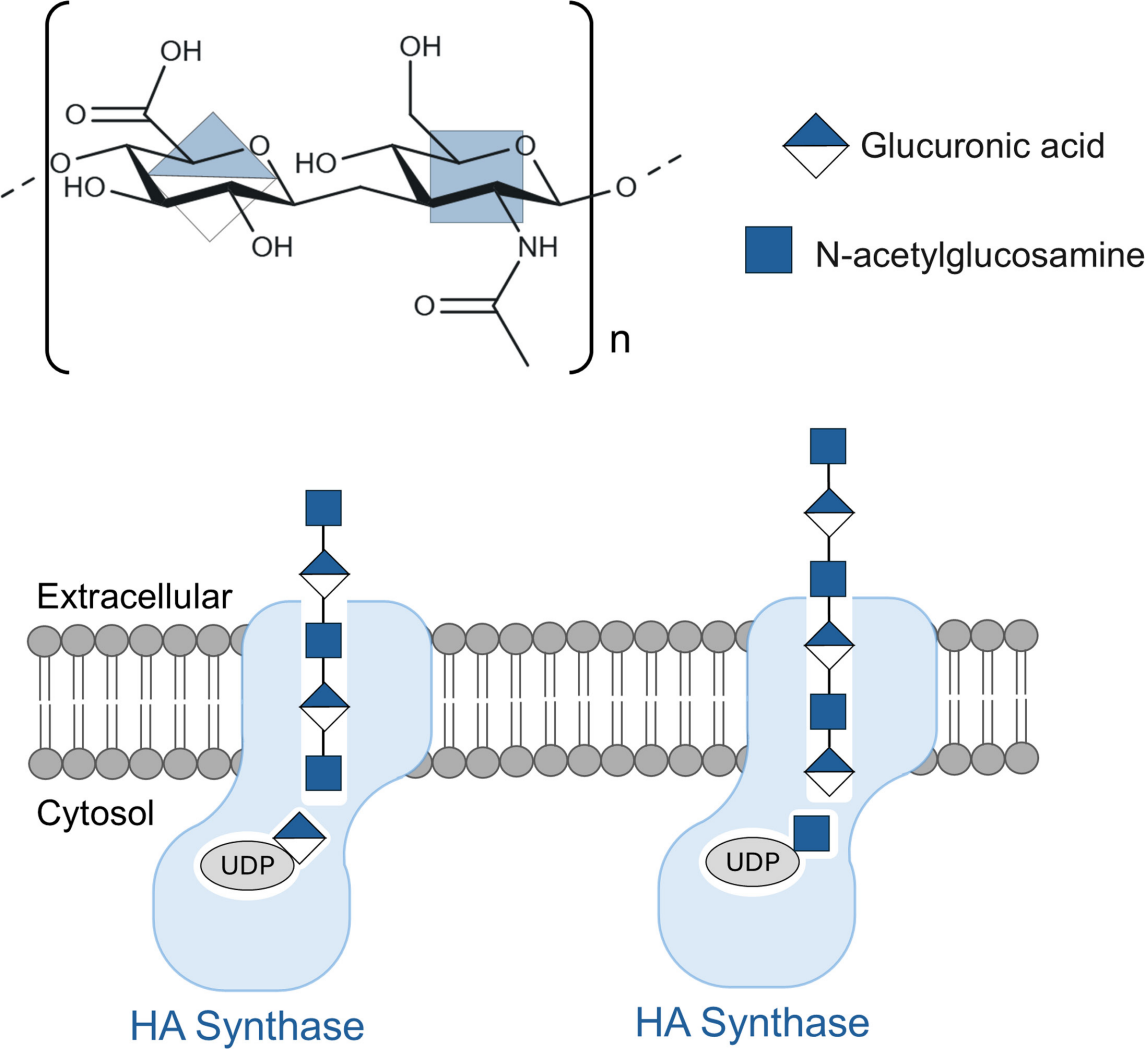
Structure, biosynthesis and translocation of the biopolymer hyaluronan. Top: Hyaluronan is a linear biopolymer composed of repeating units made of glucuronic acid (GlcA) and N-acetylglucosamine (GlcNAc). Bottom: The enzyme hyaluronan synthase (pale blue) is embedded in the cell membrane (gray) and is responsible for the biosynthesis of hyaluronan, and also for its translocation across the cell membrane. Biosynthesis involves alternately adding GlcA and GlcNAc from UDP-linked substrates inside the cell to one end of the hyaluronan, while translocation involves pushing the hyaluronan chain into the extracellular space. Stephens et al. have helped shed light on this mechanism, ensuring that GlcA is always added when GlcNAc is at the end of the polymer chain (left), and vice versa (right).

Over the past decade, researchers have clarified many aspects of hyaluronan biosynthesis, but key mechanistic questions remain. In particular, how does a single active site discriminate between the two different substrates (UDP-GlcA and UDP-GlcNAc), and how is processivity – the ability to catalyze successive reactions without releasing the substrate – maintained? Now, in eLife, Zachery Stephens, Julia Karasinska and Jochen Zimmer of the University of Virginia School of Medicine report the results of experiments on hyaluronan biosynthesis in a virus called *Chlorella* that provide answers to some of these questions (Stephens et al., 2026; Figure 1).

The researchers used cryo-electron microscopy and enzyme assays to investigate the interaction between hyaluronan synthase and its substrates. Their results indicate that the enzyme distinguishes between UDP-GlcA and UDP-GlcNAc via a two-step recognition mechanism: the substrate first binds in a preliminary ‘proofreading’ position, where it is inspected, before being repositioned into the catalytic pocket so that GlcA or GlcNAc can be added to the growing biopolymer. Moreover, the efficient addition of GlcA requires the enzyme to be primed (that is, a sugar molecule must already be bound to the enzyme’s active site). The enzyme is usually primed by GlcNAc, which is a monosaccharide, but – somewhat surprisingly – Stephens et al. found that it can also be primed by two disaccharides – cellobiose and chitobiose. This reveals an unexpected flexibility in hyaluronan biosynthesis.

It is thought that class 1-NR hyaluronan synthases are structurally similar among different vertebrate species, indicating that the mechanism of hyaluronan biosynthesis is likely shared across vertebrates. However, there are notable differences between Class 1 R and Class 1-NR enzymes. For example, Class 1 R hyaluronan synthases from *Streptococci* bacteria have been shown to function as homodimers, with one member of the dimer positioning the acceptor chain, while the other binds the incoming donor substrate (Blackburn et al., 2018). This is in stark contrast to the way that Class 1-NR hyaluronan synthases function.

Future studies are needed to investigate Class 1 R hyaluronan synthases in more detail. The results of such studies, combined with the latest findings of Stephens et al., will lead to a deeper understanding of hyaluronan biosynthesis and could have applications in biomaterials production and hyaluronan-based therapies for wound healing and tissue repair.
